# Mechanisms of sex hormones in autoimmunity: focus on EAE

**DOI:** 10.1186/s13293-020-00325-4

**Published:** 2020-09-07

**Authors:** Ninaad Lasrado, Ting Jia, Chandirasegaran Massilamany, Rodrigo Franco, Zsolt Illes, Jay Reddy

**Affiliations:** 1 School of Veterinary Medicine and Biomedical Sciences, University of Nebraska-Lincoln, Lincoln, NE 68583 USA; 2 CRISPR Therapeutics, Cambridge, MA USA; 3 Department of Neurology, Odense University Hospital, University of Southern Denmark, Odense, Denmark

**Keywords:** Sex hormones, Autoimmunity, EAE, MS, T cells

## Abstract

Sex-related differences in the occurrence of autoimmune diseases is well documented, with females showing a greater propensity to develop these diseases than their male counterparts. Sex hormones, namely dihydrotestosterone and estrogens, have been shown to ameliorate the severity of inflammatory diseases. Immunologically, the beneficial effects of sex hormones have been ascribed to the suppression of effector lymphocyte responses accompanied by immune deviation from pro-inflammatory to anti-inflammatory cytokine production. In this review, we present our view of the mechanisms of sex hormones that contribute to their ability to suppress autoimmune responses with an emphasis on the pathogenesis of experimental autoimmune encephalomyelitis.

## Introduction

The normal function of the immune system is to protect organisms against invading pathogens. When such a response is directed against self-tissues, autoimmunity may ensue. However, healthy individuals can have signatures of autoimmune response as evidenced by the detection of low levels of antibodies and T cells against autoantigens that may reflect formation of natural antibodies or idiotypic networks [1–4]. Autoimmune diseases (AIDs) are clinically manifested when autoimmunity leads to tissue damage disrupting the functions of affected organs [5, 6].

AIDs are generally noted to be the leading causes of deaths in young to middle-aged women in the USA [7]. Estimates indicate a large variation in both the incidence (less than 1 per 100,000 persons to more than 20 per 100,000) and prevalence (less than 5 per 100,000 to more than 500 per 100,000) of these diseases [8]. Approximately 50 million Americans may have some form of an autoimmune disease and of these, more than 75% are women [7]. The chronic nature of many of these diseases such as multiple sclerosis (MS) can significantly impact medical costs and quality of life [8].

MS is a chronic inflammatory and demyelinating disease of the central nervous system (CNS), and it affects approximately 2.5 million people worldwide showing a female preponderance (2 to 3:1). Within the USA alone, MS affects approximately 400,000 people with 10,000 new cases diagnosed annually [9–11] resulting in the loss of ~ 2.5 billion to the economy [12, 13]. While, the disease can be seen in people of any age, it is commonly diagnosed in the age group of third to fifth decades. Although, no known causes are identified, it is commonly believed that a combination of genetic susceptibility and environmental factors trigger the disease-onset [9, 11]. Traditionally, four types of MS have been identified. These include relapsing-remitting MS (RRMS), secondary progressive MS, primary progressive MS, and progressive-relapsing MS (PRMS) [14], with RRMS being the most common (~ 85%) and PRMS the rarest of all (~ 5%) [11]. A recent classification emphasizes combination of active or inactive, and/or stable or progressive nature of the disease course [15]. The pathological diversity of lesions in the white and grey matter with differential mechanistic signatures provides an additional layer to the variable clinical phenotypes [16, 17]. Given this complex nature, it is a challenge to study the pathogenetic events in humans, and therefore, various animal models of experimental autoimmune encephalomyelitis (EAE) are routinely used in MS research.

EAE can be induced in a wide-range of species (rodents: rabbits, rats, and mice; and non-rodents: monkeys and pigs) [14, 18–22]. The two hallmarks of EAE are inflammation and demyelination, and the disease is typically mediated by autoreactive T cells [23, 24]. While EAE-induction by active immunization involves the use of myelin antigens or their immunogenic peptides in complete Freund’s adjuvant (CFA), the disease can be transferred to naïve animals by adoptively transferring myelin-reactive T cells. Three main myelin antigens have been identified to induce EAE, namely myelin basic protein (MBP), proteolipid protein (PLP), and myelin oligodendrocyte glycoprotein (MOG), and their disease-inducing peptides are also identified. These include MBP 1-11 that induces EAE in B10.PL or PL/J mice (H-2^u^); PLP 139-151-induced EAE in SJL mice (H-2^s^) and MOG 35-55-induced EAE in C57BL/6 mice (H-2^b^) [14, 25]. Of these models, sex differences have been well noted with the PLP 139-151-induced EAE in SJL mice. In this model, while females show chronic relapsing-remitting paralysis, the disease-course is restricted to the monophasic form in male mice [26]. These phenotypes resemble some of the clinical features of MS making the SJL model of EAE to be helpful for studying sex differences in the CNS autoimmunity [26]. Here, we review the salient features of sexual dimorphism of AIDs with an emphasis on the role of T cells in the pathogenesis of EAE.

## Sexual dimorphism in the occurrence of infectious diseases vs. AIDs

**Fig. 1:**
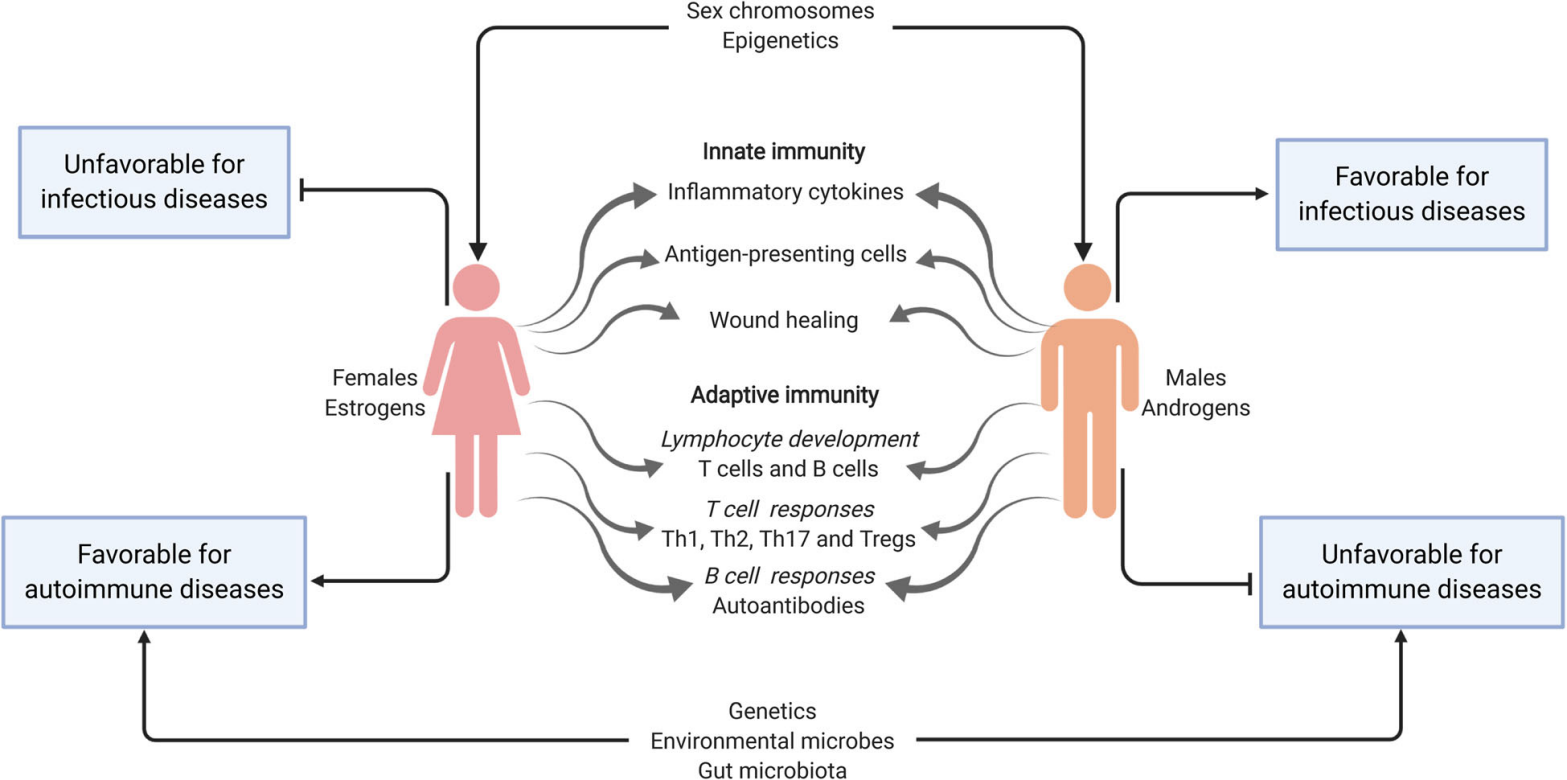
Sexual dimorphism with the disease occurrence, and its underlying potential immune mechanisms. It is generally believed that males are more prone to infectious diseases than females, but the latter group have a preponderance to develop autoimmune diseases. These phenotypes are shown with elbow arrows (favorable), and arrows with inhibitory lines (unfavorable). The hormonal environments in females (estrogens) and males (androgens) have been shown to influence both innate and adaptive immune cell functions. Additionally, hormonal actions on immune cells in the respective sexes can potentially be influenced by transcriptome profiles in the sex chromosomes and epigenetic modifications. Nonetheless, genetic susceptibility and exposure to environmental microbes, including alterations in the gut microbiota, if any are still the key players to trigger AIDs, but their outcomes can be modulated by sex hormones

It has been known for a long time that susceptibility to various diseases differs by sex. While males are more susceptible than females to viral, bacterial, and parasitic infections, the tendency to develop autoimmune diseases is higher in females than males [27] (Fig. 1).

### Infectious diseases

Females are generally more resistant than males to viral infections due to the higher antibody production [28], especially during the period between puberty and menopause [27], but the conflicting reports may question this notion. While males appear to contract certain viral infections at a higher rate—such as human immunodeficiency virus, west Nile virus, hepatitis B virus, influenza virus, and Hantavirus [28, 29]—females with the same viral load as males can be at a higher risk of developing acquired immune deficiency syndrome [30]. Similarly, during the 2009 H1N1 avian influenza pandemic in Canada, women were found to be at two- to six-fold higher risk of dying than men [31]. Conversely, emerging evidence suggests that mortalities are more common in males than female individuals affected with coronavirus disease-19 that can be ascribed to other confounding factors such as smoking and behavioral changes [32–34]. Generally, women are known to mount higher anti-viral immune responses than men which may be beneficial to clear the virus, but prolongation of such a response can lead to increased disease-severity [31, 35]. For bacterial infections however, males were found more susceptible than females to *Mycobacterium tuberculosis* (M.tb), *Helicobacter pylori*, *Coxiella burnetii*, *Pseudomonas aeruginosa*, and *Salmonella typhimurium* infections [36–40]. Additionally, the proportion of adult males found to have symptomatic M.tb infections was two-fold higher than in females [36]. Conversely, women are more likely than men to survive from sepsis [41]. Females have a lower incidence of malaria than males [42] and experimentally, female mice also were found to be more resistant than males to *Plasmodium chabaudi* infection [43]. These data suggest that sex differences may vary from disease to disease of infectious origin.

### AIDs

It is well conceived that most autoimmune diseases are more prevalent in females than males [44, 45]. This phenomenon has been well documented especially with AIDs mediated by autoantibodies such as Sjögren’s syndrome (female to male ratio of 16:1), systemic lupus erythematosus (SLE) (7:1), Hashimoto’s thyroiditis (19:1), and Grave’s disease (7:1), in which, about 80% of the patient population was female [46]. In the middle tier of diseases, which includes rheumatoid arthritis (RA) (3:1) and MS (2:1), the sex distribution has been 60–75% in women relative to men [46]. In fact, a study involving Danish cohorts revealed the risk for developing MS was increased more than two-fold in females, whereas in males, the disease remained unchanged over a period of 25 years [47]. Likewise, neuromyelitis optica spectrum disorder (NMOSD) is also characterized by a high female predominance and the disease-outcomes can also be influenced by the sex [48]. Interestingly, this difference is much higher in NMOSD associated with AQP4-antibodies, and less in seronegative NMOSD without pathogenic autoantibodies [49, 50]. However, for other diseases such as inflammatory bowel disease and type 1 diabetes (TID), the prevalence rates are similar for both sexes [51]. Conversely, Guillain-Barre syndrome appears to be occurring at equal or higher rates in males than females [51], whereas myasthenia gravis shows a female predominance in the early-onset as opposed to a male predominance in the late onset of the disease [52]. Likewise, myocarditis is more frequently reported in young men than their female counterparts [53]. Of note, male patients with later onset MS have a higher risk for faster disability progression suggesting that sex-differences may also be seen in the disease course [54].

Furthermore, occurrence of AIDs appears to be influenced by the reproductive cycles in affected individuals. For example, pre-pubertal cases of MS are extremely rare, with only 3–5% cases reported in individuals younger than 18 years of age. The finding that sexual dimorphism is seen mostly in post-pubertal women suggests that puberty is a critical risk factor [55]. For example, the female-to-male ratio for SLE is found to be 2–6:1 prior to puberty (9–14 years for boys and 8–13 years for girls), as opposed to 9:1 after puberty (≥ 15 years for boys and ≥ 14 years for girls) [56]. Additionally, disease severity can be influenced by pregnancy, as shown with MS, where the clinical signs of the disease are suppressed during pregnancy, especially during the third trimester. However, the risk of MS relapse is increased in the first 3 months of post-partum and returns to the pre-pregnancy level by 6 months after delivery [57, 58]. In the case of RA however, symptoms can be low or completely suppressed during gestation, whereas women with SLE often have exacerbated symptoms during pregnancy [56]. While, these observations point to a possibility that the sex hormones may determine the clinical outcomes of AIDs, primary triggers of these diseases remain largely unknown.

### Factors that influence the development of AIDs

Two major factors have been implicated in the induction of AIDs. These include genetic susceptibility and exposure to environmental factors and the readers may find excellent reviews on these topics elsewhere [59, 60]. Furthermore, transcriptome profiles of sex chromosomes, specifically X, and epigenetic variations also appear to influence the occurrence of autoimmunity (Fig. 1). One such transcript is KDM6a where the animals deficient for this gene were found resistant for the development of EAE [61]. Other potential candidates include Forkhead box P3 (FoxP3) and Toll like receptor (TLR) 7 [62]. Likewise, epigenetic modifications (DNA methylation, histone modifications, chromatin remodeling, and non-coding RNAs) at major histocompatibility complex (MHC) loci may influence sex differences in MS [51, 63] (Fig. 1). Additionally, polymorphisms in the interferon (IFN)-γ and interleukin (IL)-12 receptor β genes were noted with sex differences in susceptibility to MS [64, 65]. Deficiency of the Fas/CD95 death receptor was associated with decreased apoptosis of inflammatory cells in the CNS with enhanced EAE severity. Such an association was also seen in women with MS [66], suggesting that the cellular responses might be different between sexes.

Additionally, it has been recently shown that the sex differences in autoimmunity can be influenced by the gut microbiota (Fig. 1). For example, specific pathogen-free non-obese diabetic (NOD) mice show a female preponderance to develop TID, but the germ-free mice lose such a bias [67]. Furthermore, gut flora differ between sexes, a trend reversed by male castration suggesting that androgens can influence the gut microbiota [67]. Likewise, colonization by commensal microbes led to elevated serum testosterone levels and protection of male NOD mice from developing TID [68]. Importantly, transfer of gut microbes from adult males to immature females altered the microbiota in females leading to reduced islet inflammation and autoantibody production and protection from TID occurring in conjunction with increased testosterone levels [68]. These data suggest that the gut microbiota can be an important determinant of the outcomes of sexual dimorphic nature of autoimmune diseases in those affected. In support of this preposition, microbiota composition revealed diverse microbial populations in association with chronic-progressive and chronic relapsing-remitting type of paralysis as evaluated in two mouse strains namely, C57Bl/6 and SJL mice [69]. However, existence of sex-specific altered microbiota, if any that can potentially contribute to the sex bias in EAE phenotypes, needs further investigations. Taken together, the data indicate that the immune microenvironments in males and females might be uniquely influenced by sex hormones.

## Immune mechanisms of sex hormones

### Expression of sex steroid receptors in immune cells

Physiologically, estrogens are responsible for female sexual characteristics, similar to androgens in males [70]. Estrogens include estrogen (E1), estradiol (E2), and estriol (E3), of which E3 is produced only during pregnancy [71]. Their effects are mediated through estrogen receptor alpha (ERα) and estrogen receptor beta (ERβ) through the formation of homodimers or heterodimers. ERα has been detected in dendritic cells (DCs), monocytes, macrophages, natural killer (NK) cells, mast cells, B cells, and T cells [72–77]. Even though CD4 T cells express more ERα than ERβ, CD8 T cells and monocytes express low amounts of both ERs. On the contrary, B cells express higher amounts of ERβ than ERα [78].

Androgens mediate their effects predominantly by binding to androgen receptors (AR) located intracellularly [79], but they also can be expressed in a non-classic form on the cell surface [80]. Several immune cells like neutrophils, macrophages, B cells, and T cells have been shown to express AR [79, 81]. In thymic T cells, only classic AR has been detected, whereas both forms have been noted in the splenic T cells [82]. Likewise, while both macrophages and B cells can express classic AR, non-classic AR is expressed only in macrophages [83]. Since most terminally differentiated immune cells express sex hormone receptors, their functionalities can be potentially modulated by sex hormones.

### Effect of sex hormones on innate immune cells

Several reports indicate significant differences in the innate immune responses between sexes (Fig. 1). For example, healthy female macaques have increased counts of most leukocyte subpopulations in their peripheral blood than their male counterparts [84]. Similarly, healthy female mice have higher numbers of leukocytes in the pleural and peritoneal cavities than do male mice [85]. Circulating NK T cells can also be more numerous in healthy women than men [86]. Male healthy mice, however, appear to have more neutrophils than do females [87]. Such variations also have been noted in the ability to respond to microbial products. For example, in the airway inflammation model of asthma, greater numbers of macrophages and DCs were found to migrate from lungs to the draining lymph nodes in females as compared to males [88]. Human monocytes from males after lipopolysaccharide (LPS) stimulation can produce more of IL-1β, tumor necrosis factor (TNF)-α, and IL-12 than those from females [89]. Similarly, compared to female neutrophils, male neutrophils release greater amounts of TNF-α in response to LPS stimulation. This hyper-responsiveness of male neutrophils to LPS has been suggested as a potential mechanism in making males more susceptible than females to sepsis [90]. Furthermore, higher levels of TLR 7 detected in females compared to males can have implications in their ability to respond to virus infections, because TLR-7 is involved in the recognition of single-stranded viral RNA molecules [91].

### Effects of sex hormones on antigen-presenting cells

Most antigen-presenting cells express both ERα and ERβ [74, 92]. Estrogens can regulate the functions of monocytes/macrophages and DCs in various ways **(**Fig. 1). For example, E2 inhibits expression of IL-1, IL-6, and TNF-α in activated macrophages [93]. DCs pretreated with E2 can suppress antigen-presenting functions by enhancing their ability to produce the anti-inflammatory cytokines IL-4 and IL-10 [94]. However, it also has been reported that E2, acting via ERα, can promote differentiation of DCs [92]; the E2-treated DCs have superior antigen-presenting function with increased major histocompatibility complex (MHC) class II expression [95]. Similar effects also were noted with testosterone-treated macrophages [96]. Although male mice appear to have lower numbers of Langerhans cells (LC) than female mice, androgens can influence DC development [97]. Topical application of testosterone or its metabolite dihydrotestosterone (DHT) can result in a significant decrease in the density of LCs in both normal females and orchiectomized males [98]. However, DHT appears not to promote granulocyte macrophage colony-stimulating factor-driven DC differentiation [92]. Furthermore, estrogen or progesterone can activate macrophages and promote wound healing through angiogenesis and tissue remodeling [99]. Androgens also can modulate inflammatory responses during acute wound healing, as evidenced by the observation that castration or blockage of androgens can result in suppressed recruitment of macrophages [100, 101], as well as the experimental observation that AR-deficient mice show accelerated wound healing [79] (Fig. 1). These observations suggest that the innate immune functions can be modulated by estrogens or androgens similarly.

### Effect of sex hormones on adaptive immune cells

Adaptive immune responses are mediated by B cells and T cells. While some of the common lymphoid progenitors originated in the bone marrow can be educated within bone marrow to become B cells, some progenitors go to thymus and mature to become CD4 or CD8 T cells. T cells and B cells recognize self-antigens in the corresponding primary lymphoid organs. While strong recognition of self-antigens leads to the death of immature lymphocytes by negative selection, weak recognition favors positive selection of developing lymphocytes, indicating that the lymphocytes present in the peripheral repertoires must have seen the self-antigens. Conversely, if the self-antigens are not expressed in the generative lymphoid organs, then the developing lymphocytes can escape central tolerance. This has been clearly demonstrated in the case of PLP 139-151 as the naïve repertoire of SJL mice contain a significant proportion of PLP 139-151-reactive T cells [102]. Mechanistically, this phenomenon has been ascribed to the thymic expression of truncated form of PLP, called DM-20 isoform that contains a deletion in the coding region, representing the motif, PLP 139-151 [102–104]. Furthermore, in addition to repressive effects on lymphopoiesis, estrogens and testosterone can directly modulate the expression of autoimmune regulator (AIRE) protein that has a pivotal role in the thymic expression of self-antigens [105]. While estrogen suppresses AIRE via epigenetic changes [106, 107], androgens promote AIRE’s expression, an effect that can be abolished by castration [106, 108]. Whether enhanced expression of AIRE in the male thymus can be directly related to their low susceptibility to autoimmune diseases needs further clarifications.

Additionally, sex hormones have been shown to modulate lymphocyte development (Fig. 1). AR can inhibit T cell development in the thymus, as castrated animals exhibit thymic enlargement and increased numbers of lymphocytes that can be reversed by androgen-replacement therapy [83, 109, 110]. E2 has been shown to decrease B cell lymphopoiesis, since pregnancy levels of estrogens have been correlated with both a significant reduction in B cell numbers and activity of B lymphocyte precursors in the bone marrow [111]. Experimentally, formation of B cells was reduced in the bone marrow of mice treated with E2, while castration or ovariectomy led to increase in B lymphopoiesis ER-dependently [112, 113]. In addition, E2 can dampen B cell receptor (BCR) signals and favor the generation of marginal zone B cells and survival of autoreactive B cells [114, 115]. Similar suppressive effects were noted with androgen on B cell development. Assessment of B cell progenitors in the bone marrow of castrated mice revealed a dramatic increase in late pro-B cell levels, leading to an increase in the numbers of peripheral B cells, but to a lesser degree in pre-B and immature B cell populations [116, 117]. Estrogens can block T cell development and cause thymic atrophy in an ERα-dependent manner [118].

As to the peripheral repertoires, both human and macaque females appear to possess a higher number of circulating CD4 T cells, including CD4/CD8 ratios, than males [89, 119]. Likewise, human peripheral blood CD4 T cells from females produce relatively higher levels of the T-helper (Th) 1 cytokine, IFN-γ, than from males [120]. As to MS, although autoantibodies contribute to the disease pathogenesis, no sex-specific variations have been noted with antibodies in affected individuals. However, the peripheral repertoires of female humans and non-human primates can contain a relatively high proportion of activated B cells [84, 121], suggesting that lymphocyte responses can be potentially dictated by the inherent production of hormones specific to each sex.

### Effects of sex hormones on the effector lymphocyte responses

Sex hormones have been shown to exert anti-inflammatory effects (Fig. 1), and therapeutically, estrogens and DHT and their derivatives have been used in various diseases (Table 1). Specifically, as to MS, reduced brain lesions and relapse rates were noted with estrogen therapy accompanied with reduced inflammatory cytokines (Th1 and TNF-α) [122, 123]. Likewise, DHT treatment was associated with decreased fatigue and increased gray matter volume with a corresponding decrease in CD4 T cell infiltrates and IL-2 production, and increase in TGF-β1 secretion [124, 125]. Experimentally, low doses of estrogens have been shown to stimulate Th1 responses, whereas high doses equivalent to pregnancy levels can promote Th2 response in primary cultures [126, 127]. Estrogens also can stimulate the production of regulatory T cells (Tregs) by upregulating the expression of FoxP3 [128, 129], and other non-FoxP3-expressing Treg subsets such as B regulatory cells (Bregs), CD8^+^CD122^+^ Treg cells, and CD11b^+^ CD206^+^ ARG-1^+^ M2 like macrophages, among others [130]. EAE mice treated with E2 or E3 show reduced disease severity through inhibition of Th1 and Th17 cytokine production with a corresponding increase in Th2 cytokines [126, 131]. Similarly, testosterone also ameliorates EAE severity with a Th2 bias, as androgen-treated T cell lines, as opposed to untreated cultures, secrete a lower amount of IFN-γ compared to IL-10 [132–134]. Although testosterone appears not to promote differentiation of murine Treg cells, high testosterone and low estrogen conditions may promote skewing of Th1/Th17 responses toward Treg cells [135]. Recent reports suggest that males possess high frequencies of innate lymphoid cells (ILC) 2, and IL-33 produced from mast cells facilitate induction of non-pathogenic Th2 rather than encephalitogenic Th17 cytokines in the females [136]. But determination of antigen-specificity of these Th subsets has remained a major challenge in the field.

**Table 1:** Therapeutic effects of estrogen and DHT and their derivatives in various autoimmune disease conditions

Disease	Estrogen/its derivatives		DHT/its derivatives	
	Humans	Animal models	Humans	Animal models
Multiple sclerosis	Reduced Th1 response and TNF-α levels with a shift towards Th2 (IL-5, and IL-10) and reduction in lesions in the brain and relapse rate [122, 123, 168]	Enhanced B-reg and T-regs, higher serum IgG1 levels, reduced Th1, Th17 response with a shift towards Th2, as evidenced by increased IL-5 (males) and IL-10 levels, with decreased IFN-γ, TNF-α, IL-2, IL-6, IL-17, and IL-23 levels [130, 131, 169, 170]	Reduced DTH response, increased NK cells, increased TGF-β1 and decreased IL-2 levels, decreased fatigue, increased gray matter volume and decreased CD4^+^ T cell infiltrates [124, 125, 135]	Significant decrease in EAE severity, with skewness of Th1/Th17:T-reg ratio towards T-reg, and a shift towards Th2 response (increased IL-10) and decreased IFN-γ level [132, 135, 165–167]
Rheumatoid arthritis	Patients with high serum E2 showed reductions in VPS, AI [171]	Significant reduction in alkaline phosphatase, TNF-α, IL-1β, IL-6 and anti-type-II collagen autoantibody levels, and reduced disease severity [172–174]	Improved clinical signs with increased serum testosterone levels and CD8^+^ T cells, with decreased CD4^+^:CD8^+^ ratio, reduction in tender joints [175, 176]	Decreased autoantibody generation and joint inflammation, reduction in TNF-α and PGE-2 with reduced inflammatory infiltrates [73, 177, 178]
Systemic lupus erythematosus	No significant benefits were noted	No significant benefits were noted	Reduced disease severity, restoration of normal serum testosterone levels with reduced hematologic and serologic abnormalities [179–181]	Reduced disease severity with increased survival rate with no autoantibody formation [182]
Sjögren’s syndrome	No significant benefits were noted	No significant benefits were noted, but has been shown to offer some level of protection against Sjögren’s syndrome-like disease	Reduced ESR rates, increased testosterone levels offering disease protection, reduced dry-eyes and dry-mouth symptoms [183, 184]	Reduced lymphocyte infiltrations and reversal of autoimmune sequeale in lacrimal gland [185–187]
Hashimoto’s thyroiditis	Not tested	Not tested	Inverse correlation between testosterone and thyroid autoimmunity, improved thyroid secretory function [188]	Reduced disease incidence and pathology, and drastic reduction in thyroglobulin autoantibodies [189]
Crohn’s disease	Not tested	Not tested	Improved CDAI with reduced serum CRP, increased hemoglobin level, and reduced inflammation [190, 191]	Not tested
Psoriasis	Not tested	Not tested	Normal serum testosterone levels, improved disease score, reduced CRP and improved obesity [192]	Not tested
Type-I diabetes	Not tested	Not tested	Improved glycemic control with reduced fasting glucose and HbA_1c_ [193]	Not tested
Graves’ disease	Not tested	Not tested	Not tested	Amelioration of disease severity with a shift from Th1 to Th2 response, reduction in IL-2, IFN-γ and increase in IL-4, IL-10, TGF-β, IL-35, and attenuation of thyroid oxidative injuries [194, 195]
Autoimmune cholangitis	Not tested	Not tested	Not tested	Decreased pathology with lesser CD4^+^ liver-infiltrating T cells, reduced expression of CXCL-9, CXCL-10, and IL-17 with increased serum testosterone concentration [196]
Autoimmune orchitis	Not tested	Not tested	Not tested	Reduced disease severity, reduction in CD4^+^ T cells and accumulation of macrophages in testis, with significant increase in T-regs. Substantial decrease in MCP-1, TNF-α, IL-6, IL-2, and IFN-γ [197]

*VPS* visual analogue pain scale, *AI* articular index, *DTH* delayed type hypersensitivity, *PGE*-*2* prostaglandin-E_2_, *ESR* erythrocyte sedimentation rate, *CDAI* Crohn’s disease activity index, *CRP* c-reactive protein, *HbA*_*1c*_ hemoglobin A1c

**Fig. 2:**
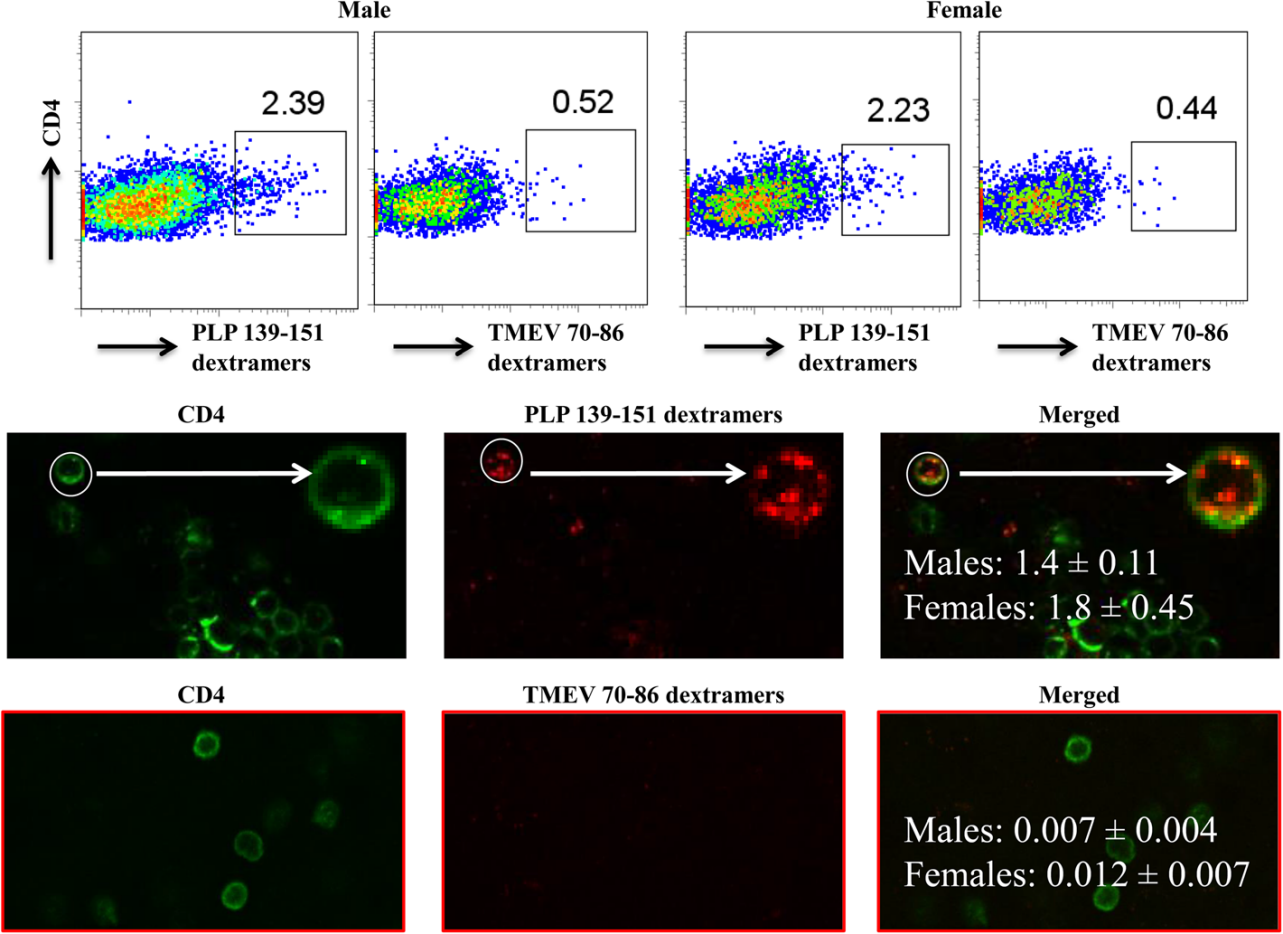
Enumeration of PLP 139-151-specific CD4 T cells in the CNS infiltrates from EAE mice*.* Male and female SJL mice were immunized with PLP 139-151, and brains and spinal cords were harvested from EAE-mice that showed paralytic signs. Mononuclear cells isolated from these tissues were stained with PLP 139-151 (specific) or control (Theiler’s murine encephalomyelitis virus [TMEV] 70-86) dextramers and the dextramer^+^ CD4^+^ cells were then analyzed. Representative flow cytometric plots are shown (top panel). By establishing in situ dextramer staining technique using LSCM, PLP 139-151-specific, CD4 T cells were analyzed in the brains harvested from male and female mice (bottom panel). CD4 T cells, green; dextramers, red; merged (circles, dext^+^ CD4^+^ T cells; insets represent enlarged views of dext^+^ CD4^+^ T cells). Original magnification × 1000; bar = 20 μm. Mean ± SEM values are shown (*n* = 3)

In our research, we made efforts to understand the cellular basis for sex bias in the occurrence of EAE in SJL mice by testing the hypothesis that the EAE-phenotypic differences between sexes are due to defects in antigen-specific, CD4 T cell responses. To this end, we created MHC class II (IA^s^) tetramers and dextramers for PLP 139-151 that can detect antigen-specific T cells with a high degree of specificity and sensitivity [137]. By enumerating the precursor frequencies of PLP-specific CD4 T cells flow cytometrically, we noted that the lymph node cells derived from male and female SJL mice responded equally to PLP 139-151, suggesting no defect in their ability to respond to self-antigens. We have also verified this phenomenon for an environmental microbe-derived epitope that cross-reacts with PLP 139-151 [138]. Furthermore, dextramer staining analysis of CNS infiltrates also did not reveal any significant variations between sexes with PLP-specific T cells as evaluated by flow cytometry (Fig. 2, top panel). Next, we established a novel in situ dextramer staining method to localize PLP-specific CD4 T cells in the brains of EAE mice by laser scanning confocal microscopy (LSCM) [139]. By evaluating brains obtained from male and female mice affected with EAE, we found the PLP dextramer^+^ cells to be scattered all through the tissues with equal proportions in both male and female mice, ruling out defects in the migration of antigen-specific T cells into the CNS (Fig. 2, bottom panel). Finally, T cells harvested from the brains of EAE mice and the T cell cultures stimulated with PLP 139-151 in vitro showed comparable expression of most of the positive and negative regulators of T cell activation in both male and female mice (unpublished observations). Based on these findings, we envision a scenario in which equal numbers of PLP-reactive, pathogenic T cells infiltrate into the brains in both male and female SJL mice, but their survivability may differ between sexes raising a question whether differences exist in the biochemical pathways between DHT and estrogen.

## Biochemical mechanisms of sex hormones

Sex hormones mediate their cellular functions through both the genomic/nuclear and nongenomic/membrane signaling pathways, with the expected end result being transcriptional regulation [140, 141] that may affect cell proliferation or cell death [142–144]. For example, in breast cancer cells, E2 stimulates cell growth by augmenting transition from G1 to S phase, leading to activation of cyclin-dependent kinase and retinoblastoma protein phosphorylation [145, 146]. Whereas other groups have also demonstrated that E2 is capable of inducing apoptosis in breast and prostate cancer cells, thymocytes, monocytes, macrophages, neuronal cells, and T cells [147–150]. Similarly, androgens also can regulate apoptosis in breast and prostate cancer cells, human renal tubular leukemic and primary cells, including monocytes and macrophages and T cells [151–153]. Recently, autophagy-associated cell death has been described that involves the upregulation of autophagy flux, its machinery and the accumulation of autophagosomes [154]. A relationship has been shown recently between sex hormones, apoptosis, and autophagy. For example, pregnancy levels of E2 and progesterone exert stimulatory effects on autophagy in mammary epithelial cells by suppressing mammalian target of rapamycin (mTOR) activation that occurs in association with apoptotic cell death [155]. Additionally, E2 may regulate transcription factors targeted by autophagy, miRNAs, and histone modifications [156]. Likewise, E2 was shown to inhibit osteoblast apoptosis by promoting autophagy via the mTOR pathway [157]. But, less is known about androgens, and they were shown to promote prostate cancer cell growth through the induction of autophagy, in part through the production of reactive oxygen species [158]. Because both autophagy and apoptosis are well-controlled biological processes that play important roles in tissue homeostasis and disease, dissecting the cross-talk between the two, if any in the context of sex hormones, may lead to identification of molecules that affect both processes [159, 160].

To address the above theme, we established an in vitro system to determine the mechanistic basis for DHT-mediated effects in autoreactive T cells, since DHT has been successfully used to treat EAE. Unexpectedly, we noted that DHT reduced the proliferative responses to PLP 139-151, but the effects were not selective, since both proliferating and non-proliferating cells were equally affected [161]. Likewise, using MHC class II dextramers, we failed to note any immune deviation toward Th2 phenotype in antigen-specific T cells; rather, cells capable of producing all major inflammatory cytokines (Th1 and Th17), including Th2 cytokines, were reduced in DHT-treated cells. We also showed that DHT-mediated effects involved the induction of cell death, which also was associated with autophagy in autoreactive T cells [161]. Although our data did not support the notion that DHT-mediated effects accompany the appearance of IL-10-producing cells [132–134], production of IL-10 by non-T cell sources in vivo or in mixed T cell cultures in response to DHT-treatment cannot be discounted. Previous reports indicate that DHT can ameliorate EAE when administered either during induction or in the effector phase of the disease process [132, 134]. Our observation that DHT induces cell death of both proliferating and non-proliferating T cells may mean that the DHT-mediated effects might have occurred due to cell death. Importantly, we have also demonstrated that cell death can occur in conjunction with autophagy in DHT-treated cells [161], suggesting that common signaling cascades, or crosstalk, may exist between the two processes. Although dissecting this complexity is a challenge, using model systems that are deficient for apoptosis and autophagy machineries, such as caspase-3- and ATG-deficient mice, may be helpful. These studies may then provide avenues to identify molecules responsive to DHT that can affect both apoptosis and autophagy processes.

## Perspectives and significance

As discussed above, autoimmune diseases are more prevalent in females than males and such a discrepancy also exists in the animal models, as shown with PLP 139-151-induced EAE in SJL mice [60, 138]. Essentially, PLP-reactive T cells generated in males can induce EAE in males comparable to the EAE-phenotype in females induced by cells generated in the female SJL mice [138]. Conversely, cells from males can induce only mild disease in females [138], suggesting that the microenvironment of recipients may determine the EAE-outcomes. By investigating the underlying mechanisms, we had previously noted that the EAE-resistant, male B10.S mice possess higher frequencies of Treg cells specific to PLP 139-151 than SJL mice, and depletion of Treg cells enabled B10.S mice to develop severe EAE [162, 163]. While these observations provide a cellular basis for EAE-susceptibility and EAE-resistance phenotypes, male hormones appear to play a critical role in the suppression of EAE. In support of this notion, a number of studies [124, 125, 132, 136, 164–166] indicate therapeutic benefits of testosterone by ameliorating the EAE-severity or clinical remissions in MS patients that are accompanied with increased gray matter volume, reduced Th1/Th17 inflammatory cytokines (IFN-γ, IL-2, and IL-17A), skewness of Th1/Th17:Treg ratio toward Tregs, shift of immune response toward Th2 type (IL-10), increased NK cell populations, and significant reductions in CNS infiltrations containing CD4 T cells [124, 125, 132, 135, 164, 165, 167]. Based on our observations with DHT [161], we did not recognize the phenomenon of immune deviation from pro- to anti-inflammatory cytokine switch; rather, DHT was found to suppress T cell responses regardless of their antigen-specificity that involve apoptosis and/or autophagy as the possible underlying mechanisms [161]. Additionally, we performed a few pilot experiments and determined that estrogens mediate effects similar to DHT (data not shown). Whether all sex hormones mediate their functions through common pathways such as apoptosis and autophagy is currently unknown. Proving this concept to be true may then widen the applications of sex hormone-dependent molecules as drug targets for a range of diseases, including metabolic syndromes, aging, and osteoporosis. Such discoveries also may potentially reduce the need to use small molecules like selective androgen receptor modulators. As a result, it may be possible to minimize side effects observed with sex hormones.

## Data Availability

Not applicable.

## Abbreviations

AIDs: Autoimmune diseases
AIRE: Autoimmune regulator
AR: Androgen receptors
BCR: B cell receptor
Bregs: B regulatory cells
CFA: Complete Freund’s adjuvant
CNS: Central nervous system
DCs: Dendritic cells
DHT: Dihydrotestosterone
E1: Estrogen
E2: Estradiol
E3: Estriol
EAE: Experimental autoimmune encephalomyelitis
ERα: Estrogen receptor alpha
ERβ: Estrogen receptor beta
FoxP3: Forkhead box P3
IFN: Interferon
IL: Interleukin
ILCs: Innate lymphoid cells
LC: Langerhans cells
LCSM: Laser scanning confocal microscopy
LPS: Lipopolysaccharide
M.tb: *Mycobacterium tuberculosis*
MBP: Myelin basic protein
MHC: Major histocompatibility complex
MOG: Myelin oligodendrocyte glycoprotein
MS: Multiple sclerosis
mTOR: Mammalian target of rapamycin
NK: Natural killer
NMOSD: Neuromyelitis optica spectrum disorder
NOD: Non-obese diabetic
PLP: Proteolipid protein
PRMS: Progressive-relapsing multiple sclerosis
RA: Rheumatoid arthritis
RRMS: Relapsing-remitting multiple sclerosis
SLE: Systemic lupus erythematosus
Th: T helper
TID: Type I diabetes
TLR: Toll like receptor
TMEV: Theiler’s murine encephalomyelitis virus
TNF: Tumor necrosis factor
Treg: T regulatory cells
